# Biopriming of Durum Wheat Seeds with Endophytic Diazotrophic Bacteria Enhances Tolerance to Fusarium Head Blight and Salinity

**DOI:** 10.3390/microorganisms10050970

**Published:** 2022-05-05

**Authors:** Adel Hadj Brahim, Manel Ben Ali, Lobna Daoud, Mouna Jlidi, Ismahen Akremi, Houda Hmani, Naser Aliye Feto, Mamdouh Ben Ali

**Affiliations:** 1 Laboratory of Microbial Biotechnology and Enzymatic Engineering (LBMIE), Center of Biotechnology of Sfax (CBS), University of Sfax, Road of Sidi Mansour km 6, P.O. Box 1177, Sfax 3018, Tunisia; manel.benali@gmail.com (M.B.A.); daoud.m@gmail.com (L.D.); jlidimanno@yahoo.fr (M.J.); asmahen.akremi@gmail.com (I.A.); houda_enis@yahoo.fr (H.H.); 2 Astrum Biotech, Business Incubator, Center of Biotechnology of Sfax (CBS), University of Sfax, Road of Sidi Mansour km 6, P.O. Box 1177, Sfax 3018, Tunisia; 3 OMICS Research Group, Department of Biotechnology, Vaal University of Technology, Vanderbijlpark 1911, South Africa; naserf@vut.ac.za

**Keywords:** endophyte, bacillus, seed biopriming, bioprotection, biotic and abiotic stress, salt tolerance, *Triticum turgidum L. var durum*

## Abstract

There is growing interest in the use of bio inoculants based on plant growth-promoting bacteria (PGPB) to promote plant growth under biotic and abiotic stresses. Here, we provided a detailed account of the effectiveness of a number of endophytic PGPB strains, isolated from the roots of the halophyte *Salicornia brachiata* in promoting durum wheat growth and enhancing its tolerance to salinity and fusarium head blight (FHB) disease. *Bacillus* spp. strains MA9, MA14, MA17, and MA19 were found to have PGPB characteristics as they produced indole-3-acetic acid, siderophores, and lytic enzymes, fixed free atmospheric nitrogen, and solubilized inorganic phosphate in vitro. Additionally, the in vivo study that involved *in planta* inoculation assays under control and stress conditions indicated that all PGPB strains significantly (*p* < 0.05) increased the total plant length, dry weight, root area, seed weight, and nitrogen, protein, and mineral contents. Particularly, the MA17 strain showed a superior performance since it was the most efficient in reducing disease incidence in wheat explants by 64.5%, in addition to having the strongest plant growth promotion activity under salt stress. Both in vitro and in vivo assays showed that MA9, MA14, MA17, and MA19 strains were able to play significant PGPB roles. However, biopriming with *Bacillus subtilis* MA17 offered the highest plant growth promotion and salinity tolerance, and bioprotection against FHB. Hence, it would be worth testing the MA17 strain under field conditions as a step towards its commercial production. Moreover, the strain could be further assessed for its plausible role in bioprotection and growth promotion in other crop plants. Thus, it was believed that the strain has the potential to significantly contribute to wheat production in arid and semi-arid regions, especially the salt-affected Middle Eastern Region, in addition to its potential role in improving wheat production under biotic and abiotic stresses in other parts of the world.

## 1. Introduction

Biotic and abiotic stresses have been reported to severely limit crop growth and yield [1]. Salinity is one of the most important abiotic factors affecting crop production. Several investigations have indicated that climate change could potentially increase the salt level at different latitudes [2]. In Tunisia, salt-affected areas are quickly escalating due to intensive exploitation by drainage and irrigation [3], the intrusion of saline water into arable lands and the use of chemical fertilizers and pesticides.

To reduce the negative effects of salt (NaCl) on plants, different tools have been developed such as plant genetic engineering [4], osmopriming with different chemical factors such as aerated solution of ascorbate, salicylic acid, kinetin and CaCl_2_ [5] and also the use of plant growth-promoting bacteria (PGPB) [6,7].

PGPBs are known to stimulate plant growth via various mechanisms [8,9,10]. To improve crop productivity, these PGPB strains can be applied using different methods. The first one is the direct inoculation of pure [11] or mixed [12] PGPB strains or the co-application of PGPB and chemicals such as N-fertilizers [13] and the second is seed biopriming before the germination phase [14]. The latest one is a method used to enhance seed germination either under optimal or stress conditions [15]. Interestingly, the positive impact of priming was found to be more pronounced under stress than control samples [16], a finding that yet needs further investigation to understand the underlying mechanism. Relatively, seed biopriming is considered a simple, low-cost and low-risk technique as compared to other techniques that allow glycophytes to grow under salt-stress conditions [17,18,19]. This technique was successfully used in many crops including hot pepper [20,21], lettuce [22], maize [23], okra [24], tomato [25,26], pea [27], pepper [28], milkthistle [29], and soybean [30].

The application of PGPB strains via seed biopriming through soaking grains for a specified period of time in a bacterial suspension initiated the physiological processes related to seed germination, while radicle and plumule do not emerge [31]. Seed biopriming was reported to improve the tolerance of crop plants to salt-stress. To achieve this effect, several PGPB strains have been used to date; however, strains belonging to *Bacillus* species have been most frequently utilized. For instance, *Bacillus* species have successfully been used as priming agents in crops such as potato [32], radish [33], rice, mung bean, and chickpea [34].

Halophytes are able to grow under salt stress using different physiological and biochemical mechanisms, such as the secretion of small molecules as endogenous gasotransmitters [35], genome-wide identification of microRNAs [36], salt glands secretion, alteration in ion homeostasis, and the detoxification of reactive oxygen species (ROS) and alterations in membrane composition [37] in addition to their interactions with the associated PGPB [38]. However, there have been many published works on the effect of the rhizospheric and endophytic bacteria associated with the halophytes grown under natural saline conditions [39,40,41]. Hence, in the current study, the authors reported the evaluation of biopriming seeds with beneficial plant growth-promoting endophytic bacteria (PGPB) associated with halophyte *Salicornia brachiata* on the potential of bioprotection effect against fusarium head blight in durum wheat, and the evaluation of the effect of these PGPB isolates for improving tolerance to salt stress and promoting durum wheat growth.

## 2. Methods

### 2.1. Isolation of Endophytic Bacteria

Prior to isolation, root samples of *Salicornia brachiata* collected in south-east Tunisia (34°46′16″ N, 10°48′24″ E) were washed to eliminate soil traces and then rinsed by sterile deionized water in order to selectively isolate diazotrophic bacteria. The clean roots were submerged in a solution of HgCl_2_ for 2 min and then in an alcohol solution for 15 s. In order to isolate the endophytic bacteria, the sterile roots of *Salicornia brachiata* were washed in a sterile solution of physiological water, spread on solid LB medium, and incubated at 30 °C for 24 h to verify the strict absence of microflora on the roots surface. Finally, the roots were washed several times with sterile ultrapure water and macerated in a sterile PBS solution (0.2 M, pH 7.0) and the macerate was inoculated in a culture flask containing Burk’s N-free medium with the composition (g/L): glucose, 10; KH_2_PO_4_, 0.41; K_2_HPO_4_, 0.52; CaCl_2_, 0.2; MgSO_4_/7H_2_O, 0.1; FeSO_4_/7H_2_O, 0.005; Na_2_MoO_4_/4H_2_O, 0.0025; NaCl, 10, and incubated for 7 days at 30 °C. Then, 0.1 mL aliquots of a 6-fold serial dilution of root macerate were spread on Burk’s N-free medium supplemented with yeast extract of 0.5%. After incubation for 7 days at 30 °C, the morphologically different colonies were isolated and subcultured for further analysis.

### 2.2. Assessment of the Nitrogen-Fixation (diazotrophic) Potential of the Isolates

In order to understand the nitrogen-fixing potential of the strains, the isolates were grown on nitrogen-free culture medium NFMM (g/L): malic acid, 5.0; KOH, 4.0; K_2_HPO_4_, 0.5; FeS0_4_/7H_2_0, 0.05; MnS0_4_/7H_2_0, 0.01; MgS0_4_/7H20, 0.01; NaCl, 0.02; CaCl_2_, 0.01; Na_2_Mo0_4_/4H_2_O, 0.002; bromothymol blue, 0.5% alcoholic solution and pH adjusted to 7.0. Then, in order to investigate the nitrogenase activity and estimate the N-fixing capacity of the strains, the ARA test was performed. The isolates that showed a positive and predominant growth during the first 3–4 days were selected for quantification based on the acetylene reduction method in nitrogen-free semi-solid medium. For this purpose, bacterial cultures were grown in five vials of 15 mL volumes of semi-solid NF broth medium with composition (g/L): malic acid, 5.0; K_2_HPO_4_, 0.5; MgS0_4_/7H_2_0, 0.2; NaCl, 10; CaCl_2_/2H_2_0, 0.05; agar, 1.8, and the 50 mL vials were sealed with rubber septa; 36 h after the inoculation, and a 1/10 volume (air volume) of acetylene was added. The amount of ethylene was measured every 5 h for a total of 36 h. All incubations were done at 30 °C in the dark, avoiding any movement of the vials. Ethylene was measured using a gas chromatograph equipped with a flame-ionization detector and a packed column (1.83 m long, 0.3 cm in diameter, stainless steel, packed with HayeSep N; Supelco). Calculations were based on peak area [42] and the Kjeldhal N-digestion and distillation system [43]. The selected isolates were incubated in 10 mL of NF broth in a rotary agitator (DAIHAN LabTech. Co. LTD, LSI-3016A) for 5 days at 150 rpm and 28 °C. The N content in the microbial cells was determined according to [44].

### 2.3. Effect of Physiological Conditions on the Growth of Potent N Fixing Bacteria

The effect of various growth conditions such as temperature, salt tolerance and pH on the growth of the most potent N fixers was checked in an NFMM medium. For studying the effect of temperature, potent endophytic bacteria were incubated at temperatures viz., 25, 30, 35, 40 °C for 48 h at 200 rpm in a rotary agitator (DAIHAN LabTech. Co. Ltd., Namyangju, Korea, LSI-3016A). The NFMM medium supplemented with different concentrations of NaCl (ranging from 0–400 mM NaCl) was used for salt tolerance studies and the hydrogen ion concentration in the range of 4–12 was selected for pH studies. The growth of the potent N fixing endophytic bacteria under the given growth conditions was observed by taking the optical density of the medium with a UV-1600 PC Jenway 7305 spectrophotometer.

### 2.4. Molecular Characterisation of Endophytic Bacterial Isolates

#### 2.4.1. Genomic DNA Extraction

The genomic DNA extraction of the endophytic bacterial isolates was carried out with the chemical lysis-phenol extraction method as described by [45]. After each of the cell lysis treatments (chemical lysis) in a solution of TE Buffer pH 8 which contains 10 mM Tris-HCl and 1 mM EDTA, supernatants containing DNA were centrifuged at 12,000 rpm for 10 min at room temperature to remove the cell debris as well as the beads, and the bacterial pellet was resuspended in a 500 µL lysis buffer pH 8 which contained 50 mM Tris-HCl, 10 mM EDTA, 2 mg/mL lysozyme, and incubated at 37 °C for 30 min. After this step of lysis, 45 µL of 20% SDS and 5 µL of 10 µg/mL Proteinase K were added and the reaction was conducted at 55 °C until clear and viscous solution was obtained. DNA was extracted with 1 vol of phenol, followed by a second extraction with 1/2 vol phenol–1/2 vol chloroform: isoamyl alcohol (24:1). DNA was precipitated in the aqueous phase by adding 2.5 vol of ethyl alcohol overnight at −20 °C. Samples were then centrifuged at 8000× *g* for 10 min, the supernatants were removed and the pellets containing DNA were resuspended in 100 µL 70% (*v*/*v*) cold ethyl alcohol and centrifuged for 15 min at room temperature. Each pellet was air-dried, suspended overnight under agitation in 50 µL of pure water, and pooled. RNA was eliminated from the sample by RNase treatment.

#### 2.4.2. Identification of the Selected Strains

In order to identify the selected and relevant strains, a partial sequence analysis of the 16 S rDNA was performed. PCR reaction and DNA sequencing were performed as described by [46]. 16 S rRNA gene was amplified using universal forward (5-AGAGTRTGATCMTYGCTWAC-3) and reverse (5-CGYTAMCTTWTTACGRCT-3) primers. A reaction mixture of 100 μL containing 100 ng DNA template, 400 ng of each primer, 4 μL dNTP (2.5 mM each), 10 μL 10 × Taq DNA polymerase assay buffer, and 1 μL Taq DNA polymerase (3UμL^−1^) was prepared. PCR reactions were carried out in a thermal cycler (Model ABI 2720, Applied Biosystems International, Foster City CA, USA). The PCR cycle used for amplification was as follows: 5 min at 94 °C, followed by 35 cycles of 30 s at 94 °C, 30 s at 55 °C, 2 min at 72 °C and a final extension of 5 min at 72 °C. The amplified 16 S rRNA gene was purified with a PCR purification kit Qiaquick PCR purification kit, Qiagen, India) and outsourced for sequencing (Chromus Biotech Pvt. Ltd., Bangalore, India). The sequence data were aligned with the System Software aligner and analysed to identify the bacterium and its closest neighbours using BLAST (NCBI, Bethesda, MD, USA). The partial 16 S rRNA gene sequences were deposited in GenBank data base.

### 2.5. ACC Deaminase Activity Assay

The assessment of the ACC deaminase activity was carried out by determining the content of α-ketobutyric acid released after ACC cleavage as previously reported by [47]. In brief, the bacterial cells were grown in a minimal medium containing ACC as the sole nitrogen source in a 15 mL TSB medium with the following composition (g/L): casein peptone, 17; soya peptone, 3; NaCl, 5; K_2_HPO_4_, 2.5; glucose, 2.5; final pH 7.3 ± 0.2 at 25 °C up to log phase to elicit their ACC deaminase activity. The production of α-ketobutyrate was monitored at a 540 nm absorbance wave length. The standard curve of α-ketobutyrate ranging from 0.1 to 1.0 μmol was used to calculate the ACC deaminase activity that was expressed as the amount of α-ketobutyrate generated per mg of protein per hour.

Lytic enzymes *β*-glucanase, chitinase, protease and phytase activities were assessed using *β*-glucan, chitin, milk and phytic acid, respectively, as substrates in a modified LB agar medium (1.0% enzyme substrate, 1% NaCl, 0.5% yeast extract, 0.5% peptone, 0.5% (NH_4_)_2_HPO_4_, 0.02% KCl, 0.3% MgSO_4_/7H_2_O) [48]. The isolates were spotted in the plates, incubated for 3 days at 30 °C and the halo zone was checked.

### 2.6. In Vitro Plant Beneficial Traits of Bacterial Isolates

HCN production was checked by observing the change in colour of the filter paper impregnated with 0.5% picric acid in 1% Na_2_CO_3_ [49]. IAA production was quantified by growing bacterium for 3 days in LB-broth and detected in the presence or absence of 0.5% tryptophan in culture filtrate using Salkowski’s reagent (1 mL of 0.5 M FeCl_3_ in 50 mL of 35% HClO_4_). After an incubation of 25 min at room temperature, the absorbance of pink colour was measured at 530 nm [50,51], whereas siderophore production was determined on a Chrome-Azurol S medium according to [52]. The development of a yellow–orange halo around the growth was considered as positive for siderophore production. Phosphate solubilization activity was tested by spot inoculation on Pikovskya‘s medium [53,54]. Briefly, the test isolates were inoculated in 25 mL Pikovskya’s broth and incubated for 4 days at 30 °C. The bacterial cultures were centrifuged at 15,000 rpm for 30 min. The absorbance of the developing blue colour was read at 530 nm. The production of EPS was characterized by the size of the halo produced and its slime appearance. Each strain was inoculated onto 5 mm diameter paper discs disposed in a medium (2% yeast extract; 1.5% K_2_HPO_4_; 0.02% MgSO_4_; 0.0015% MnSO_4_; 0.0015% FeSO_4_; 0.003% CaCl_2_; 0.0015% NaCl; 1.5% agar) modified by the addition of 10% of saccharose, with a pH value of 7.0. EPS production was monitored by chilled ethanol precipitation method according to [55]. Organic acid production was tested on NFMM medium (g/L): malic acid, 5.0; KOH, 4.0; K_2_HPO_4_, 0.5; FeS0_4_/7H_2_0, 0.05; MnS0_4_/7H_2_0, 0.01; MgS0_4_/7H_2_0, 0.01; NaCl, 0.02; CaCl_2_, 0.01; Na_2_Mo0_4_, 0.002; bromothymol blue, 0.5% alcoholic solution and pH adjusted to 7.0, using bromophenol blue as pH indicator [56,57]. Biocontrol activities were assessed with three *Fusarium* species and *Rhizoctonia solani* with minor modifications of the method described by Mahmood et al. [58]. All these strains of phytopathogenic fungi were taken from a selection bank of microbial strains of the Biotechnology Centre of Sfax.

### 2.7. Biocontrol Activity against Fusarium Head Blight Tested in a Greenhouse

In our present study, the sensitivity of *Triticum turgidum durum* seeds ‘Aouija’ variety, provided by the Agriculture Research Station at the Centre of Biotechnology of Sfax, to different NaCl concentrations was assessed in order to determine the sublethal dose to be applied in the subsequent experiments (in vivo and in vitro). The plant growth experiments were defined under greenhouse conditions in 9 cm diameter pots containing 1 kg/pot of natural soil added with different NaCl concentrations. Accordingly, the plant growth was affected at 50 mM NaCl and severely retarded at 125 mM NaCl, in vitro and in vivo. Thus, the later concentration (125 mM NaCl), which corresponds to the cut-off point of growth retardation in *Triticum turgidum L. var durum* in vitro, was applied in further experiments.

To investigate the ability of various bacterial isolates to suppress head blight disease in wheat, wheat seeds were disinfected by immersion in NaOCl (2%) for 3 min followed by incubation in ethanol (75%) for 2 min before rinsing 3 times in sterile distilled water. The selected bacterial isolates were grown in LB medium (Sigma Aldrich, St. Louis, MO, USA) for 24 h at 30 °C, harvested by centrifugation for 10 min at 5000× *g* and washed twice with phosphate-buffered saline (PBS, pH 7.0).

The seeds bioprimed in a 1 × 10^8^ cfu/mL bacterial suspension for 1 h were placed onto 9 cm plates covered with sterile wet filter paper and incubated for 72 h at 25 °C for germination. In order to determine the biocontrol activity of the two selected PGPB strains (*Virgibacillus halodenitrificans* MA14 and *Bacillus subtilis* MA17), pots were placed in a controlled growth chamber (Phytotron type Misa 2000 LWeiss Technik France). Thereafter, the obtained plantlets were placed in the sterile soil, prepared after three successive autoclaving cycles at 121 °C for 15 min, in a growth chamber at 25 °C under 16/8 h photoperiod and irrigated with a 1/2 Hoagland solution. One week later, the wheat seedlings were inoculated with 2 mL of *F. graminearum* Schwabe suspension (10^7^ cfu/mL).

The treatments were as follows: control 1: not bioprimed; control 2: inoculated only with *F. graminearum* Schwabe suspension; treatment 1: seed bioprimed with *Virgibacillus halodenitrificans* MA14 and inoculated with *F. graminearum* Schwabe suspension; treatment 2: seed bioprimed with *Bacillus subtilis* MA17 and inoculated with *F. graminearum* Schwabe suspension. Data for the percentage of disease incidence and severity were collected on the 30th day after sowing. Each treatment had seven replicates. The control efficiency (CE) was determined according to the formula: [CE (%) = (DIcontrol-2—DI T)/DIcontrol-2 × 100%], where DI: disease index; and T: one of the treatments [59]. 

### 2.8. Inoculum Preparation and Seed Biopriming

The PGPB strains were incubated in LB broth overnight with constant shaking at 28 ± 2 °C with 200 rpm in a Daihan-LabTech shaker model LSI-3016A. Cells were harvested by centrifugation and resuspended in normal saline to an optimum concentration (OD = 1 × 10^8^ cells cfu per mL at λ = 600 nm). Seeds of *Triticum turgidum L. var durum.* ‘Aouija’ were disinfected with HgCl_2_ (0.1%) for 2 min and rinsed twice with sterile distilled water. The bacterial suspension was added to the seeds and subjected to constant 100 rpm shaking along with a sterile carrier material (peat with 5 mm of particle size) until a thin film is formed as a proof of seeds being coated. Finally, the coated seeds were air-dried before sowing.

### 2.9. Seed Germination Assay

All coated seeds (10 per pot) with beneficial isolates were sown separately in each pot filled with treated soil. Uncoated seeds in pots filled with untreated soil were kept as a positive control. The experiment was triple-checked. Seed germination percentages were recorded after seven days.

### 2.10. Salt Stress of Potted Plants

Natural soil without sterilization was used in the pot assay. The used soil was iso-humic (Ustifluvent) with pH 7.05, EC 0.21 mS m^−1^, 4.40 g kg^−1^ organic carbon, 89 kg ha^−1^ available N (alkaline permanganate extractable). The waterproof pots were used for maintaining a humid microenvironment around the plant throughout the 5 months of the experiment. After the germination of the coated bioprimed seeds for 7 days, five plantlets per pot were placed under glasshouse conditions at 25 ± 2 °C for five months in a completely randomized design. Trial 1: control samples—plants grown without PGPB in small pots (9 cm diameter) containing natural soil (1 kg/pot); Trial 2: treated samples—the plants without PGPB were treated with 125 mM NaCl in small pots (9 cm diameter) containing natural soil (1 kg/pot); Trial 3: inoculated samples—seed biopriming with PGPB in small pots containing natural soil (1 kg/pot); Trial 4: treated and inoculated samples—the experiment was conducted by seed biopriming with PGPB in small pots containing natural soil (1 kg/pot) treated with 125 mM NaCl. 

### 2.11. Analysis of Morphological and Biochemical Plant Growth Parameters

After a 5-month-treatment, the plant growth parameters were identified. The dry weight (DW) and the total length of each plant were measured. The length of the roots and shoots and 100 seed mass was measured. The DW was recorded after drying in an oven at 70 °C for 72 h. 

The nitrogen and total protein content were carried out with the Kjeldhal and distillation method [43] and Bradford [60], respectively.

### 2.12. Ash Determination for Total Mineral Salt Content

For salt extraction, the overall ash was recorded after drying the total biomass of roots and shoots without spikes in a muffle furnace at 550 °C for 12 h according a method described with ISO 1171-1981, and the total ash was dissolved in deionized water. The salt from the supernatant was then crystallized by using isothermal evaporation at 60 °C for 12 h, and the pellet was dissolved in deionized water. The crystallization through the evaporation step was repeated until no salt crystal precipitate remained within the supernatant. All salt crystals were collected and weighed to determine the salt yield [61].

### 2.13. Statistical Analysis

The data of biochemical characterization, seed germination, plant growth parameters, protein, nitrogen, and total salt contents were subjected to Duncan’s Multiple Range Test (DMRT) using the Statistica software (ver. 10.1, StatSoft, Hamburg, Germany). For the antagonism test, the data were analysed by the Duncan’s Multiple Range Test (DMRT) using the IBM SPPSS software (ver. 21.1, SPSS Inc., Chicago, IL, USA). The significance level for all analyses was *p* < 0.05.

## 3. Results

### 3.1. Biochemical Characterization of Plant Beneficial Traits of Bacterial Isolates

PGPB strains were isolated from the roots of the halophyte *Salicornia brachiata* from a coastal area of Tunisia (34°46′16″ N, 10°48′24″ E). Among the 22 pertinent strains, four strains were retained for their diversified biological activities (nitrogen fixation, production of growth hormones, and secretion of antimicrobial substances, etc.). The selection of the relevant strains was made based on their ability to fix free atmospheric nitrogen, secrete plant growth hormones, solubilize complex minerals, and produce antimicrobial substances.

The isolates were also evaluated for their tolerance to different concentrations of salt and growth at different temperatures and pH values. We found that almost all bacterial isolates were able to grow in LB medium under all NaCl concentrations tested after 48 h (0 to 1.18 M), with an optimal growth under 340 mM NaCl for all tested strains at 30 °C and a pH of 6.5 (Supplementary Figure S2a). Therefore, all bacterial isolates were able to grow after 48 h in an NFMM medium with optimal growth under 34, 85, 170, 204 mM of NaCl for, respectively, *Bacillus pumilus* MA9, *Virgibacillus halodenitrificans* MA14, *Bacillus subtilis* MA17 and *Bacillus safensis* MA19. 

In addition, the strains were positive for *β*-glucanase (Figure S1a), protease (Figure S1b), chitinase as well as for important plant growth-promoting (PGP) properties including the production of siderophores (Figure S1c), antimicrobial compounds, and the hydrolytic potential of 1-aminocyclopropane-1-carboxylate (ACC deaminase positive) (Table 1). The tested strains were found to produce variable amounts of auxins (Table 1) ranging from 35 to 376 µg/mL. The *Bacillus subtilis* MA17 and *Bacillus safensis* MA19 produced relatively less auxin without and with tryptophan at 56/109 and 35/125 µg/mL, respectively. Particularly, *Virgibacillus halodenitrificans* MA14 was found to produce more indole acetic acid (IAA) than the rest of the tested strains and in a higher yield than the values reported in the literature [62]. The isolates also showed a phosphate-solubilizing activity, as evidenced by the formation of clear zones on Pikovskya’s medium agar plates. In the liquid Pikovskya’s medium, the bacterial isolates solubilized variable amounts of phosphates ranging from 9.8 to 47.6 µg/mL (Table 1).

The antifungal activity for the different isolates was evidenced by the formation of inhibition zones between the bacterial isolates and the fungal isolates *Rhizoctonia solani*, *Fusarium solani*, *F. oxysporum*, and *F. graminearum* (Table 1). All tested diazotrophic endophytic bacteria, except *Virgibacillus halodenitrificans* MA14, showed antagonistic effects to *R. solani*, *F. solani* and *F. graminearum*. It is worth mentioning that *Bacillus subtilis* MA17 showed the only one among the four strains tested which has antifungal activity against *F. oxysporum* (Table 1).

### 3.2. Identification of Bacterial Isolates

The analysis of the 16S rRNA gene-based sequences for the different PGPB strains was performed using BLASTn of the sequences against the NCBI’s database (http://blast.ncbi.nlm.nih.gov/) accessed on 28 June 2020.

The sequences of these bacterial isolates were submitted in the NCBI GenBank under the following accession numbers MT672745, MT672746, MT672747, MT672748 for MA9, MA14, MA17, MA19, respectively. It was, accordingly, found that the sequences of the isolates MA9, MA14, MA17, and MA19 showed 99.78, 98.25, 99.33, and 98.7% similarity with *Bacillus pumilus* SH-B9 (NZ_CP011007), *Virgibacillus halodenitrificans* A7 (MT590720), *Bacillus subtilus* HR4 (MT645613), and *Bacillus pumilus* CIP 52.67 (NR115334), respectively. 

### 3.3. Diazotrophic Potential of the Isolates

The results in Table 2 confirmed the diazotrophic activity of the PGPB strains. The four selected strains were subjected to several tests to verify their capacity for the free binding of nitrogen including the formation of a pellicle on the surface of a minimum culture medium without shaking, the capacity to change the colour of the medium towards blue by the secretion of buffer substances or towards yellow by the production of organic acids. All the tested strains showed a marked ability to fix atmospheric nitrogen as a source of nitrogen when cultured in a minimal medium.

Thus, it was of prime interest to deepen our study by estimating the ability of the selected strains to produce proteins through the reduction in acetylene to ethylene (ARA method) and the Kjeldhal nitrogen according to [42] and [43], respectively. The results indicated that all tested strains were able to grow in the minimal medium without any nitrogen supply and glucose as the sole carbon source (Table 2; Supplementary Figure S2b). The *Virgibacillus halodenitrificans* MA14 and *Bacillus subtilis* MA17 strains were the most efficient ones in terms of pellicle formation when grown in a nitrogen-free NFMM medium. Moreover, all of the strains were able to convert acetylene to ethylene after 72 h of incubation and produce nitrogen based on the ARA test and the Kjeldhal assay, respectively. The nitrogenase activities for these strains ranged from 0.452 to 3.125 nmol of C_2_H_4_ formed mL^−1^ culture media 72 h^−1^ based on the ARA assay, while it ranged from 9.458 to 51.023 mg N_2_ fixed 50 mL^−^^1^ culture media 72 h^−1^ based on the Kjeldhal assay. Therefore, the *Virgibacillus halodenitrificans* MA14 and *Bacillus safensis* MA19 have the same bromothymol colour change, but a highly significant difference (*p* < 0.05) on dinitrogen fixation. The *Virgibacillus halodenitrificans* MA14 revealed the lower N2 fixation ability and showed a significant difference (*p* < 0.05) between other strains by ARA test. In addition, the *Bacillus subtilis* MA17 isolate showed both the maximum ARA and Kjeldhal levels of nitrogen without a significant difference (*p* < 0.05) between the other strains tested by Kjeldhal test, but exhibited the lowest growth rate under the nitrogen-free medium. All these results imply that the strains had functional nitrogenase activity. 

### 3.4. Evaluation of Mechanisms Regulating Plant Homeostasis

1-aminocyclopropane-1-carboxylate activity (ACC) assays were carried out in vitro in a chemically defined medium to determine whether the different strains might play a role in regulating plant stress homeostasis.

**Figure 1:**
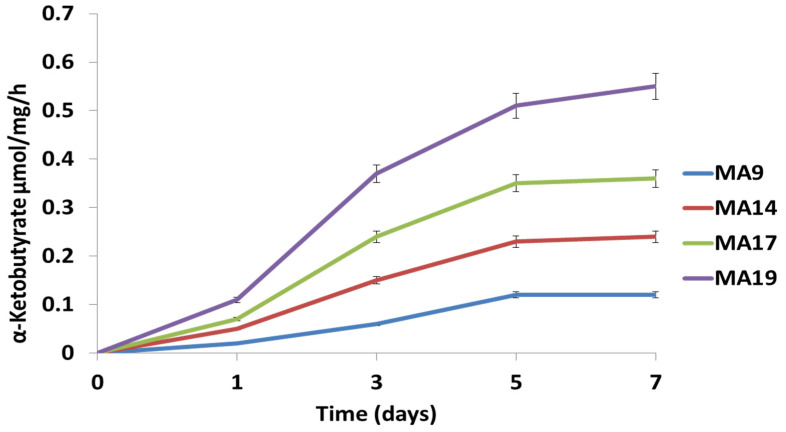
ACC deaminase activity expressed in µmol ketobutyrate mg^−1^ h^−1^ of selected endophytic bacteria on N-free medium (NFMM) supplemented with ACC (1-aminocyclopropane-1-carboxylate) as nitrogen source. Error bars show the standard deviation of the mean values of three replicates (*p* < 0.05).

In fact, the bacterial culture in NFMM medium with 5 µg/mL of ACC suggested active metabolic pathways of ACC degradation for *Bacillus pumilus* MA9, *Virgibacillus halodenitrificans* MA14, *Bacillus subtilis* MA17 and *Bacillus safensis* MA19 (Figure 1). The growth test revealed that three strains, *Bacillus pumilus* MA9, *Virgibacillus halodenitrificans* MA14 and *Bacillus subtilis* MA17 grew better in the NFMM medium supplemented with ACC than in that without it, while *Bacillus safensis* MA19 grew equally well on NFMM and NFMM + ACC media (Supplementary Figure S2b,c). In this study, all tested strains were able to produce variable amounts of ACC deaminase while the strain *Bacillus safensis* MA19 had the highest production after 7 days of incubation. As known, the ACC metabolic pathway promotes plant growth under stress conditions by reducing the ethylene concentration in plant cells. 

### 3.5. PGPB Seed Biopriming Enhanced Germination Rate under Salt Stress

**Figure 2:**
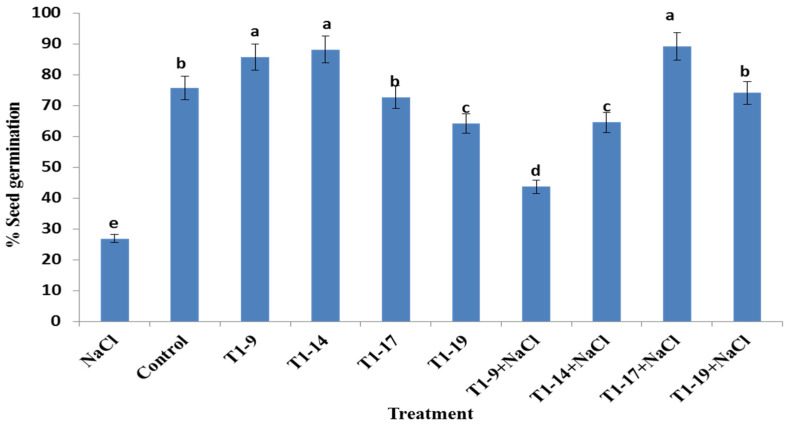
Effect of seed biopriming with PGPBs on the germination of durum wheat under control and salt stress conditions. The seeds were incubated in a suspension of 1 × 10^8^ cfu/mL bacteria on 0 and 125 mM NaCl at room temperature for 30 min. Bars represent the standard error of the mean (SEM). Different letters above the error bars indicate a significant difference at *p* < 0.05. Control, no NaCl and no PGPB; NaCl, no PGPB in salinized soil (125 mM NaCl); T1, bioprimed seed; 9, *Bacillus pumilus* MA9; 14, *Virgibacillus halodenitrificans* MA14; 17, *Bacillus subtilis* MA17; 19, *Bacillus safensis* MA19.

Regarding germination assays, we found that all of the selected PGPB strains were able to enhance wheat seed germination under salt stress as compared to the NaCl-treated samples (-PGPB, 125 mM NaCl) (Figure 2). *Bacillus pumilus* MA9, *Virgibacillus halodenitrificans* MA14, *Bacillus subtilis* MA17 and *Bacillus safensis* MA19 significantly (*p <* 0.05) improved the germination rate by 44, 65, 89 and 74% under 125 mM NaCl treatment, respectively. Additionally, *Bacillus pumilus* MA9 and *Virgibacillus halodenitrificans* MA14 improved the germination rate even without NaCl treatment (Figure 2). The authors found that seed biopriming with *Bacillus subtilis* MA17 and *Bacillus safensis* MA19 strains enhanced the germination rate of wheat seeds more than other tested strains under stressful conditions and almost all tested PGPB had certain PGPB traits, among which *Bacillus subtilis* MA17 is the only one that exhibited all of these traits (Table 1). Under untreated seeds, it was confirmed that seed biopriming with *Bacillus pumilus* MA9 and *Virgibacillus halodenitrificans* MA14 strains improved the germination rate of durum wheat more than *Bacillus subtilis* MA17 and *Bacillus safensis* MA19.

### 3.6. Biocontrol Effect of PGPB Strains against Fusarium Head Blight in Wheat

In our present study, biocontrol experiments were conducted to evaluate the bioprotection effect of two selected PGPB strains (*Bacillus subtilis* MA17 and *Virgibacillus halodenitrificans* MA14) against *F. graminearum* *Schwabe* in wheat (Supplementary Figures S3–S5). The data obtained revealed that the head blight severity was reduced in the samples inoculated by the two strains compared to the controls. It is worth mentioning that *Virgibacillus halodenitrificans* MA14 did not exhibit an in vitro antifungal activity against *F. graminearum* despite the bioprotection it rendered to the plant, which means that indirect mechanisms might have been involved (induced systemic resistance, ISR) or niche exclusion might have been involved in its in planta activity. The *Bacillus subtilis* MA17 strain provided more efficient bioprotection against the disease in the soil without fertilizer compared to *Virgibacillus halodenitrificans* MA14 with disease indexes of 36 and 76%, respectively. Moreover, *Bacillus subtilis* MA17 was found to provide both plant growth promotion and bioprotection against fusarium head blight (Table 1 and Table 3). In our work, we evidenced that, among the tested isolates, the *Bacillus subtilis* MA17 strain was the most efficient one in promoting plant growth under stress conditions and enhancing resistance to head blight disease (Table 1, Table 2, Table 3 and Table 4; Supplementary Figures S3 and S7). 

The biocontrol efficiency was also accompanied by enhanced morphological parameters of the explant such as the root length, shoot length, total length, total dry weight, total survival of plants after treatment, and disease index. Thus, seed biopriming with both *Virgibacillus halodenitrificans* MA14 and *Bacillus subtilis* MA17 provided significant resistance to head blight in wheat explants but *Bacillus subtilis* MA17 was more efficient than *Virgibacillus halodenitrificans* MA14 in terms of disease reduction efficiency (Supplementary Figures S3–S5). 

### 3.7. PGPB Seed Biopriming Improved Biomass Growth of Wheat Plants

In the in vivo experiments, 125 mM NaCl corresponding to the cut-off point of growth retardation of the *Triticum turgidum L. var durum* and the average of the optimum growth concentration of the four tested strains was chosen.

Under NaCl treatment, seed biopriming with *Bacillus pumilus* MA9, *Virgibacillus halodenitrificans* MA14, *Bacillus subtilis* MA17 and *Bacillus safensis* MA19 strains induced an increase in root length by 48, 46, 27, and 70% (Supplementary Figure S6a) and in shoot length by 15, 7, 99 and 39%, respectively, as compared to the NaCl-treated plants.

However, in comparison with untreated plants, *Bacillus safensis* MA19 decreased the root length by 71% and enhanced total length by 48%. This MA19 strain improves well the growth of shoots plant mass. On the other hand, *Bacillus subtilis* MA17 and *Bacillus pumilus* MA9 increased the root length by 21 and 130%, respectively (Supplementary Figure S6a). Additionally, *Bacillus subtilis* MA17 and *Bacillus safensis* MA19 strains offered the highest increase in total plant length compared to the untreated plants (Supplementary Figure S6b). Moreover, the majority of the PGPB used were able to offer a significant improvement of plant growth under salinity stress, as evidenced by the various agro-morphological traits (total dry weight, grain weight, root length, total length) scrutinized in the bioprimed wheat plants (Table 4; Supplementary Figures S6 and S7). 

Under non-stressful conditions, seed biopriming with the different strains increased both the total dry weight of the durum wheat cv. ‘Aouija’ plants and the mass of the 100 seeds compared to the untreated samples (Table 4 and Supplementary Figure S7). *Bacillus subtilis* MA17 offered improvement rates of 20 and 30% compared to the controls for the total dry plant mass and the mass of 100 seeds, respectively. 

Under 125 mM NaCl, all of the tested strains, *Bacillus pumilus* MA9, *Virgibacillus halodenitrificans* MA14, *Bacillus subtilis* MA17, and *Bacillus safensis* MA19, significantly enhanced the total plant dry weight by 145, 162, 341 and 166% (Table 4; Supplementary Figure S7a) and clearly improved the 100 seed mass compared to the NaCl control by 23, 27, 77, and 33%, respectively (Table 4 and Supplementary Figure S7b).

### 3.8. Effect of PGPB Treatments on Nitrogen (N) and Protein Contents of Wheat Seeds

**Figure 3:**
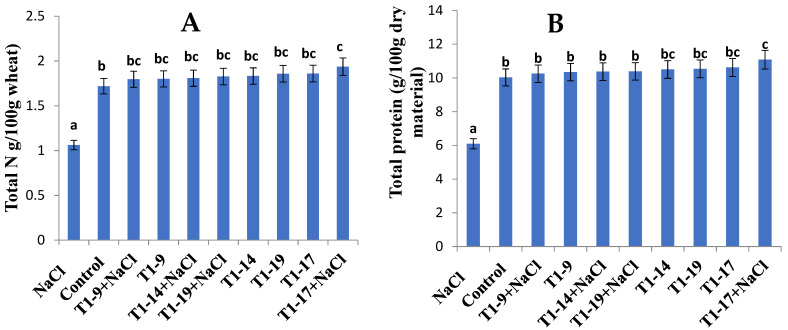
Effect of seed biopriming with PGPB strains on the wheat N-content (g/100 g wheat) (**A**) and the total protein content (g/100 g wheat) (**B**) of durum wheat plants under a different salinity regime. Bars represent the standard error of the mean (SEM). Different letters above the error bars indicate significant difference at *p* < 0.05. Control, no NaCl and no PGPB; NaCl, no PGPB in salinized soil; T1, bioprimed seed; 9, *Bacillus pumilus* MA9; 14, *Virgibacillus halodenitrificans* MA14; 17, *Bacillus subtilis* MA17; 19, *Bacillus safensis* MA19.

In the present study, the variations of the N and protein contents in wheat seeds in response to the different PGPB isolates under both stress and control conditions were monitored (Figure 3). Indeed, these data revealed an increase in N and other essential elements which were sufficiently transferred to plants by the tested diazotrophic PGPB strains (Figure 3). Both N and protein contents in wheat seeds were significantly (*p* < 0.05) enhanced, whatever the strain applied for biopriming under stress treatments compared to the uninoculated-stressed samples.

On the other hand, our findings revealed that the application of different PGPBs under stressful conditions significantly (*p* < 0.05) increased plant protein contents compared to the stressed control (Figure 3b). The highest protein content of 11.07 g 100 g^−1^ was recorded for *Bacillus subtilis* MA17 under salt stress followed by that for *Bacillus subtilis* MA17 under non-stressful conditions (10.61 g 100 g^−1^). In the non-bioprimed non-stressed control, the total protein content was 10.02 g 100 g^−1^. There was a relative increase in protein content in plants treated with *Bacillus pumilus* MA9, *Virgibacillus halodenitrificans* MA14, *Bacillus subtilis* MA17 and *Bacillus safensis* MA19 strains compared to the untreated plants by 2.29, 3.4, 10.47, and 3.6%, respectively.

### 3.9. PGPB Seed Biopriming Enhanced Total Salt Content of Wheat Plants

**Figure 4:**
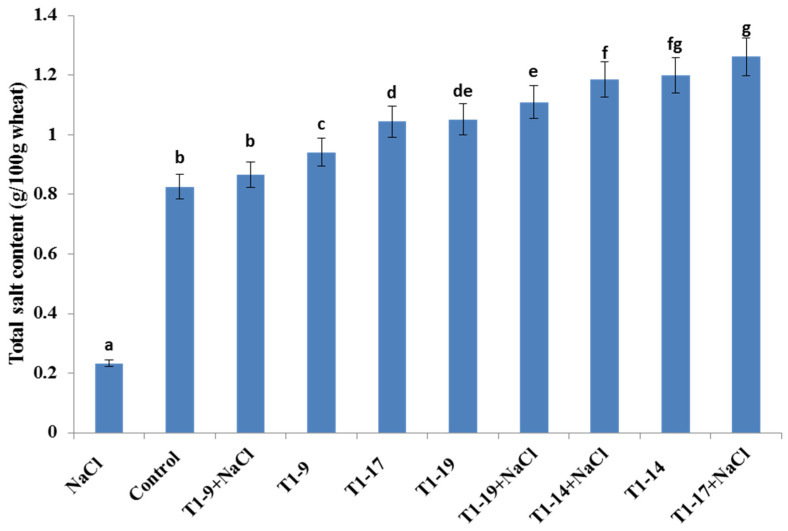
Effect of seed biopriming with PGPB strains on the total salt content (g 100 g^−1^ wheat) of durum wheat plants under different salinity regimes. Bars represent the standard error of the mean (SEM). Different letters above the error bars indicate a significant difference at *p* < 0.05. Control, no NaCl and no PGPB; NaCl, no PGPB in salinized soil; T1, bioprimed seed; 9, *Bacillus pumilus* MA9; 14, *Virgibacillus halodenitrificans* MA14; 17, *Bacillus subtilis* MA17; 19, *Bacillus safensis* MA19.

Roots and shoots of durum wheat plants constituted the fraction of the subject for the determination of the total salt content of plants. A reduction by 40% in total plant dry weight was noted in salt- treated plants compared to untreated plants (Table 4). In addition, compared to the untreated samples, the total salt content in treated plants was increased by 72% (Figure 4). Interestingly, with or without NaCl treatment, the plants bioprimed with the selected PGPBs showed an increase in their total salt content. The untreated bioprimed plants with *Bacillus pumilus* MA9, *Virgibacillus halodenitrificans* MA14, *Bacillus subtilis* MA17, and *Bacillus safensis* MA19 showed an increase in their total salt content by 303, 415, 348, and 351%, respectively, compared to the untreated samples, while in salt-treated plants, the biopriming with *Bacillus pumilus* MA9, *Virgibacillus halodenitrificans* MA14, *Bacillus subtilis* MA17, and *Bacillus safensis* MA19 resulted in 4.8, 43.6, 52.8, and 34.3% higher total salt contents compared to salt-treated plants, respectively (Figure 4). Moreover, the increase in total mineral content in plant tissues might be due to an efficient biochemical process that these selected PGPB (MA17, and MA19) strains may harbour. In fact, these PGPB strains exhibited some important traits such as the stabilization of ionic membrane permeability, the secretion of auxin, the solubilization of inorganic elements, the production of hydrolytic enzymes (*β*-glucanase, protease, chitinase), and EPS and ACC deaminase activity, under stress conditions (Figure S1 and Table 1). 

## 4. Discussion

The present study was conducted to isolate endophytic bacterial isolates from roots of halophyte *Salicornia brachiata* and their screening for potential PGPB traits in vitro to be employed for enhancing durum wheat tolerance to salt stress and fusarium head blight via seed-biopriming technology. To our knowledge, the present study is the first report to evaluate seed biopriming with halotolerant diazotrophic endophytes from roots of halophyte *Salicornia brachiata* in Tunisia, and four *Bacillus* spp. isolates were retained for their approved biological activities in vitro. The results obtained in this study showed that biopriming seeds of durum wheat with diazotrophic PGPB improved tolerance to fusarium head blight and salinity.

The idea of eliminating the use of chemical inputs in agriculture (fertilizers, stimulators, antibiotics, etc.)—which are sometimes environmentally unsafe—is slowly becoming a reality because of the emergence of microorganisms that can serve the same purpose or even perform better [63]. These microorganisms isolated from the soil and the rhizospheric part of plants, called PGPBs, are ubiquitous, safe for agricultural use and have the same characteristics as all microflora of soils. Olanrewaju et al. summarized in a review [63] that PGPBs can remove the deleterious effect of chemical inputs and promote plant growth by both direct mechanisms, including the production of auxin, ACC deaminase, cytokinin, gibberellin, nitrogen fixation, phosphorus solubilization, and the sequestration of iron by bacterial siderophores, and indirect mechanisms which refer to ACC deaminase, antibiotics, cell wall degrading enzymes, competition, hydrogen cyanide, induced systemic resistance, quorum quenching, and siderophores.

The bacteria from genus *Bacillus* is of particular importance as PGPB, given their high resistance to stressful conditions and their aptitude for plant surface colonisation and to produce a wide range of secondary metabolites [64,65] and spores. *Bacillus* spp. are known for their ability to grow under various biotic and abiotic stresses. Thus, they are considered in numerous plant studies as the dominant isolates [66,67], and are therefore recognized as an aerobic or facultative anaerobics [68]. In order to test the survival ability of the strains under severe conditions, they were incubated for long time duration under 340 mM NaCl in NFMM and we found that the strains *Virgibacillus halodenitrificans* MA14 and *Bacillus safensis* MA19 had the best ability to support stressful conditions compared to other strains (Supplementary Figure S2b). In the in vitro study, it was confirmed that all these tested strains of *Bacillus* spp. acquired one or more plant growth mechanisms. Regarding the mechanisms of action of PGPBs, it was reported that PGPB might release lytic enzymes for many applications such as the biodegradation of cellulolytic [67] or lignolytic biomass, siderophores [69], IAA and/or antibiotics [70,71] leading to pathogen growth restriction. In this work, we evidenced that, out of the tested isolates, the *Bacillus subtilis* MA17 strain was the most efficient one in promoting plant growth under stress conditions and enhancing resistance to head blight disease (Table 1, Table 2, Table 3 and Table 4; Supplementary Figures S3 and S7). 

Many studies demonstrated that antimicrobial compounds are the elicitor factors with resistance to many phytopathogenic microbes [72,73]. In this study, *Bacillus subtilis* MA17 had a significant antimicrobial activity in vitro under four phytopathogenic species, including *Fusarium graminearum* Schwabe. This *Bacillus subtilis* MA17 strain secreted one or more antimicrobial molecules which constitute elicitor factors responsible for resistance to head blight disease in wheat plants. It was reported that the double inoculation of wheat seeds with antagonistic bacteria and two phytopathogenic species of *Fusarium culmorum* isolates showed that germination and seedling vigour were generally improved in vitro [73]. *Fusarium graminearum* causes the devastating head blight of small grain cereals including wheat and barley [74]. Seed biopriming with both *Virgibacillus halodenitrificans* MA14 and *Bacillus subtilis* MA17 provided significant resistance to head blight in wheat explants but *Bacillus subtilis* MA17 was more efficient than *Virgibacillus halodenitrificans* MA14 in terms of disease reduction efficiency. This was associated with the production of diverse biologically active compounds by the *Virgibacillus halodenitrificans* MA14 and *Bacillus subtilis* MA17 ones, including siderophores, lytic enzymes as chitinase, growth hormones and a stable ACC deaminase activity. In the in vivo assay, the *Virgibacillus halodenitrificans* MA14 strain enhanced tolerance to head blight disease by 24.5%, while in the in vitro assay, *Virgibacillus halodenitrificans* MA14 does not present any activity against all strains of tested phytopathogenic fungi. This enhancing effect of *Virgibacillus halodenitrificans* MA14 is probably related to the strong production of siderophores or lytic enzymes. It is deduced that the elicitor factors enhancing resistance to phytopathogenic agents were of different natures: siderophores, antimicrobial molecules, or others. Moreover, it was reported that PGPB can indirectly improve plant growth under stress conditions through the production of siderophores, antimicrobial compounds and chitinase that are known for their role in bioprotecting host plants from various invaders [75,76].

In this study, all tested strains were able to produce variable amounts of ACC deaminase and *Bacillus safensis* MA19 had the highest production after 7 days of incubation. It is known that plant growth-promoting rhizobacteria (PGPR) with 1-aminocyclopropane-1-carboxylic acid (*ACC*) *deaminase* activity have the potential to promote plant growth and development under adverse environmental conditions [77]. In this context, numerous PGPB strains that are able to produce ACC deaminase enzymes were found to promote plant growth under stress [77,78,79].

Several approaches have been reported to promote plant growth under natural and stressful conditions such as genetic engineering transformation via agrobacterium-mediated transformation [80] and inoculation with PGPB [81,82]. Our study focuses on the “seed biopriming” approach as solid inoculants are applied and we see that soil application requires large amounts of inoculants, which is economically demanding for commercial farms. Solid inoculants are easy to apply, while liquid ones need careful handling during transportation and after field inoculation. Among these, the treatment of the seeds (priming) is performed either by microbial (biopriming), physical or chemical agents [5] during storage or just before the sowing. The chemical agents zinc, phosphate, boron, and manganese priming were mainly used in micronutrient-deficient soils, which improve the assimilation and retention of these microelements in plants and seeds [83]. In most cases, micronutrient application through seed treatment has unexpectedly performed better than other application methods [84], since chemical priming is not strongly recommended given the undesirable effects of chemicals inputs on ecological aspects of environments and thus human health. Interestingly, appropriate conditions were standardized for an efficient colonization of the seed by the bacterial inoculum during the seed priming process [85] to allow the multiplication of PGPB in the seed as well as in the spermosphere even before the sowing [86]. Thereafter, PGPB can invade the inner seed tissue, and trigger important biological activities in the seed. After successful seed colonization with PGPB, [13] confirmed that seed biopriming by *Trichoderma* spp. with varied concentrations of chemical nitrogenous fertilization clearly indicated that root infection with *Trichoderma* spp. was significantly enhanced in three different soils. Plant beneficial bacteria also have to compete with the local bacteria and other soil organisms in the root zone for colonization [87] and under severe competitive conditions; PGPB also secrete siderophores and lytic enzymes to limit the growth of plant pathogens [88] and metabolites [89]. They also release certain antibiotic compounds for better colonization [70].

On the other hand, under the “seed biopriming” approach, germination might occur rapidly and plant growth performance can be promoted [90]. Before germination, PGPB can invade the inner seed tissue, and trigger important biological activities in the seed. In the present study, the authors found that seed biopriming with *Bacillus subtilis* MA17 and *Bacillus safensis* MA19 strains enhanced the germination rate of wheat seeds more than others tested strains under stressful conditions and almost all tested PGPB had certain PGPB traits among which *Bacillus subtilis* MA17 is the only one that exhibited all of these traits. Under untreated seeds, it was confirmed that seed biopriming with *Bacillus pumilus* MA9 and *Virgibacillus halodenitrificans* MA14 strains improved the germination rate of durum wheat more than *Bacillus subtilis* MA17 and *Bacillus safensis* MA19. This significant improvement in germination rate in untreated seeds is explained with the high growth ability of *Bacillus pumilus* MA9 and *Virgibacillus halodenitrificans* MA14 strains under low salt concentration. Meanwhile, the high sensitivity to salt limits the growth and thus the biological activity and germination rate.

Moreover, the majority of PGPB we used were able to offer a significant improvement of plant growth under salinity stress as evidenced by the various agro-morphological traits scrutinized in the bioprimed wheat plants. These results are in accordance with previous reports describing the ability of different PGPB strains, such as those belonging to the *Bacillus* genus, to provide abiotic-stress tolerance in various plant species including potato [32], radish [33], rice, mungbean, and chickpea [34]. 

It was reported that biopriming winter wheat seeds with the bacterial quorum sensing signal N-hexanoyl-L-homoserine lactone (C6-HSL) shows potential to improve seed germination, plant growth, and seed productivity [91]. The significant improvements seen in wheat growth, productivity and yield structure are explained by healthier and more robust plants resulting from the direct or indirect effects of C6-HSL on seed germination, plant growth and maturation. In fact, it has been concluded that these tested PGPB strains can promote wheat growth through some important traits such as the secretion of auxin, the solubilization of inorganic phosphate, production of hydrolytic enzymes (*β*-glucanase, protease, chitinase), EPS and ACC deaminase activity, under stress conditions.

Clearly, salt stress can inhibit the metabolic pathways underlying nitrogen assimilation that is an essential element required for the growth of durum wheat plants as well as protein biosynthesis. On the other hand, these data of biopriming in durum wheat revealed an increase in N and other essential elements, which are transferred to plants in sufficient quantity by the tested diazotrophic PGPB strains. Unfortunately, the agricultural soils underwent the rapid degradation and depletion of nitrogenous matter responsible for plant growth following excessive use by field crops. This threat was overcome by different techniques such as intercropping [10], the use of PGPB, and natural phytoremediation [92]. In fact, the result of total nitrogen content in non-bioprimed and bioprimed salt-treated plants and untreated plants clearly showed that the bacterial treatment before germination significantly improved the plant’s capacity to assimilate nitrogen from the soil under salt stress. The highest total N contents were recorded in *Bacillus subtilis* MA17-bioprimed and *Bacillus subtilis* MA17-bioprimed salt-treated plants, respectively. Such an increase in N content by PGPB relied on their capacity to excessively colonize the plant roots under stress constraints. Nitrogenase activity was recorded in all PGPB treatments and the data obtained revealed an interplay between the nitrogen and protein contents. Such an increase in these elements in plant organs may be due to N_2_ fixation [93] and the P solubilization capacity of the selected PGPB strains [94], thereby allowing plant uptake via several processes such as acidification, chelation, and ion-exchanger actions [95]. The promotion in plant growth and N/protein levels in bioprimed-plants clearly reflects the ability of bacterial isolates to offer a higher nutrient flux to the plant, leading to enhanced plant biomass [96]. In a related study, it was demonstrated that the N-content in wheat shoot increased by 1.7–2.43% following pretreatment with PGPB strains as compared to untreated control samples [97]. 

Moreover, the increase in total mineral content in plant tissues might be due to an efficient biochemical process that these selected PGPB (MA17, and MA19) strains may harbour. Among ion transporters in plants, the sodium transporter, also known as the high-affinity K^+^-transporter (HKT1) which belongs to the HKT gene family, is involved in the uptake of sodium through the roots and its recirculation from shoot to root [98]. It has been confirmed that the secretion of bacterial EPS in the rhizospheric soil of plants enhanced the chelation of a harmful concentration of Na^+^. It was reported that HKT1 is permeable to K^+^/Na^+^ and systemically alleviates salinity stress through the upregulation of gene expression in shoots and downregulation in roots. 

This HKT1 gene transcription with higher levels in shoot recirculate Na^+^ from shoot xylem to root phloem in which lower HKT1 gene transcription does not allow sodium to return in the plants. It was confirmed that the exopolysaccharide (EPS) secreted with the AK-1 strain ameliorates the binding of free Na^+^ from the soil and thus alleviates the toxic effect of Na^+^ on the soybean plants [98]. 

## 5. Conclusions

Both in vitro and in vivo studies showed that *Bacillus pumilus* MA9, *Virgibacillus halodenitrificans* MA14, *Bacillus subtilis* MA17, and *Bacillus safensis* MA19 strains are PGPBs—all of which can promote wheat growth through some important PGP traits such as the secretion of auxin, the solubilization of inorganic phosphate, the production of hydrolytic enzymes, (*β*-glucanase, protease, chitinase), EPS and ACC deaminase activity, under stress conditions. Among all these bacterial isolates, the *Bacillus subtilis* MA17 showed the best vegetative growth-promotion effect in *Triticum turgidum L. var durum* in terms of seed germination, total length, total dry weight, seed mass, and nitrogen and protein content. However, biopriming with *Bacillus subtilis* MA17 offered the highest bioprotection against fusarium head blight (FHB), plant growth promotion, and salinity tolerance. Therefore, it was concluded that *Virgibacillus halodenitrificans* MA14 can protect wheat plants against FHB by another PGP traits than antimicrobial compounds production. 

Eventually, it is interesting to check the potential of the *Bacillus subtilis* MA17 to contribute in wheat production in the arid and semi-arid regions—especially in the salt-affected Middle Eastern Region, in addition to its potential role in improving plant production under biotic and abiotic stresses in other parts of the world.

## Figures and Tables

**Table 1:** Plant growth-promoting traits and other properties of selected isolates from coastal saline soil in Tunisia.

PGPB Strain	PGP Properties						Inhibition Halot of Antifungal Activity (cm)			
	IAA + Tryp *	IAA-Tryp *	Phosphate Solubilization *	SID	ACC Deam. *	EPS	*Rhizoctonia solani*	*Fusarium solani*	*Fusarium* *oxysporum*	*Fusarium* *graminearum*
MA9	202	86	47.6	+	+++	++++	5	1.5	-	1
MA14	376	216	9.8	++++	++	+	-	-	-	-
MA17	109	56	37.4	++	+	++++	6	4	2.5	5
MA19	125	35	29	+++	+++	+	5	1	-	4.5

* ACC deaminase activity was measured after 7 days of bacterial growth. IAA-Tryp and IAA + Tryp expressed in µg/mL. Phosphate solubilization expressed in µg/mL. (+) low (++) medium (+++) high (++++) very high activity. All experiments were performed in duplicate.

**Table 2:** Plant growth-promoting attributes of salt-tolerant endophytic bacteria at 2% NaCl concentration.

Strain	Pellicle Formation	N-Free Growth Medium	Blue Bromothymol Colour Change	Dinitrogen Fixation (mg N_2_ Fixed 50 mL^−^^1^ Culture Media 72 h^−1^)	ARA (nmol of C_2_H_4_ Formed mL^−^^1^ Culture Media 72 h^−^^1^)
MA9	+	++	Green blue	24.548 b	2.348 b
MA14	++	+++	Yellow	9.458 b	0.452 a
MA17	++	++	Blue	51.023 b	3.125 b
MA19	+	+++	Yellow	37.149 b	2.428 b

(+) low, (++) medium, (+++) high growth rate. All biochemical analyses were carried out in triplicate. Means in the columns followed by the same letters indicate no significant difference (*p* ˂ 0.05) by Duncan’s multiple range test (DMRT).

**Table 3:** Bioprotection efficiency of PGPB strains against wheat fungal wilt caused by *Fusarium graminearum Schwabe*.

Treatment	Disease Index (%)	Bioprotection Efficiency (%)
Control-1	0 ± 0	-
Control-2	89.58 ± 0.38 a	-
Treatment-1	76 ± 0.18 b	24.5
Treatment-2	36 ± 0.89 c	64.5

Control-1 (not inoculated with any microbe); Control-2 (inoculated only with *F. graminearum*); Treatment-1 (inoculated with *Virgibacillus halodenitrificans* MA14 and *F. graminearum Schwabe*); and Treatment-2 (inoculated with *Bacillus subtilis* MA17 and *F. graminearum Schwabe*). Values with the different letters within the same column are significantly different at *p* < 0.05 according to the Duncan multiple range test (DMRT). Numbers following the “±” are standard errors.

**Table 4:** Influence of seed biopriming with PGPB on growth parameters of wheat plants under control and saline (in vivo) conditions.

Seed with	NaCl mol·L^−1^	Root and Shoot Dry Weight (g/plant)	Root Length (cm)	Shoot Length (cm)	Total Length (cm)
Control samples *	0	1.68 a	7 cd	42.57 ab	49.57 b
MA9	0	2.55 b	16.14 e	38.57 ab	54.71 c
MA14	0	3.09 b	7 cd	61 bc	68 e
MA17	0	5.04 b	8.5 d	63.64 d	72.14 f
MA19	0	3.61 b	5 ab	63.14 bc	68.14 e
Treated samples *	0.125	1.01 a	3.71 a	37.87 a	42.28 a
MA9	0.125	2.48 b	5.42 abc	43.45 bc	48.87 b
MA14	0.125	2.65 b	20.14 f	35.14 a	55.28 c
MA17	0.125	3.45 b	4.66 ab	75.34 d	80 g
MA19	0.125	2.69 b	6.28 bcd	52.72 bc	59 d

* Control samples (- NaCl, - PGPB) and * treated samples (+ NaCl, - PGPB). Different letters after the values indicate significant difference at *p* ˂ 0.05.

## Data Availability

All data generated or analysed during this study are included in this published article and in the Supplementary Materials. The permanent link of the sequencing data of the four strains which are subject of this study are as follows: MA9/ https://www.ncbi.nlm.nih.gov/nuccore/MT672745.1?report=GenBank. MA14/ https://www.ncbi.nlm.nih.gov/nuccore/MT672746.1?report=GenBank. MA17/ https://www.ncbi.nlm.nih.gov/nuccore/MT672747.1?report=GenBank. MA19/ https://www.ncbi.nlm.nih.gov/nuccore/MT672748.1?report=GenBank. (accessed on 28 June 2020).

## Supplementary Materials

The following supporting information can be downloaded at: https://www.mdpi.com/article/10.3390/microorganisms10050970/s1. Figure S1: Production of *β*-glucanase (A), proteases (B) and siderophores (C) in vitro by bacterial isolates 1: *Bacillus pumilus* MA9, 2: *Virgibacillus halodenitrificans* MA14, 3: *Bacillus subtilis* MA17 and 4: *Bacillus safensis* MA19 after 7 days of growth at 28 ± 2 °C. The formation of halo zone around the colonies shows the positive activity. Figure S2: (A) Salinity tolerance assay of selected strains on LB medium after 48 h with different concentration of NaCl; (B) Bacterial growth assay after 7 days of selected bacteria on N free medium (NFMM) and (C) Bacterial growth assay after 7 days of selected bacteria on NFMM+ACC as the only available nitrogen source. These three bacterial growth assays were carried out under 30 °C and pH 6.5. Error bars show the standard deviation of the mean values of three replicates (*p* < 0.05). Figure S3: Effect of seed biopriming with PGPB’s strains on fusarium wilt suppression expressed in root length (A) and shoot length (B) following inoculation with *Fusarium graminearum* in seedling explants of durum wheat in a controlled culture chamber. Figure S4: Effect of seed biopriming with PGPB strains on fusarium wilt suppression expressed in total length (A) and total dry weight (B) following inoculation with *Fusarium graminearum* in seedling explants of durum wheat in a controlled culture chamber. Figure S5: Effect of seed biopriming with PGPB strains on fusarium wilt suppression expressed in survival of explants (A) and disease index (B) following inoculation with *Fusarium graminearum* in seedling explants of durum wheat in a controlled culture chamber. Figure S6: Effect of seed biopriming with PGPB strains on root length (A) and total length (cm) (B) of durum wheat plants without and with 125 mM NaCl treatment. Figure S7: Effect of seed biopriming with PGPB strains on total dry weight (A) and 100 seed mass (B) of durum wheat plants without and with 125 mM NaCl treatment.

## Abbreviations

ACC: aminocyclopropane-1-carboxylate; AcdS: ACC deaminase enzymes; ARA: acetylene reduction assay; cfu: colony forming unit; EPS: exopolysaccharides; FHB: fusarium head blight; HCN: hydrocyanic acid or hydrogen cyanide; IAA: indole acetic acid; ISR: induced systemic resistance; HKT1: high-affinity K^+^ -transporter; LB: Luria broth; NFMM: nitrogen-free minimum medium; OD: optical density; PBS: phosphate-buffered saline; PGPB: plant growth-promoting bacteria; ROS: reactive oxygen species; TSB: tryptic soy broth.
