# Supplementary material for: Biopriming of Durum Wheat Seeds with Endophytic Diazotrophic Bacteria Enhances Tolerance to Fusarium Head Blight and Salinity
Source: Microorganisms. 2022 May 5;10(5):970. doi: 10.3390/microorganisms10050970 (PMC9147649; doi:10.3390/microorganisms10050970)
Supplement: Supplementary file 1 [file microorganisms-10-00970-s001.zip › microorganisms-1684723-supplementary.pdf]

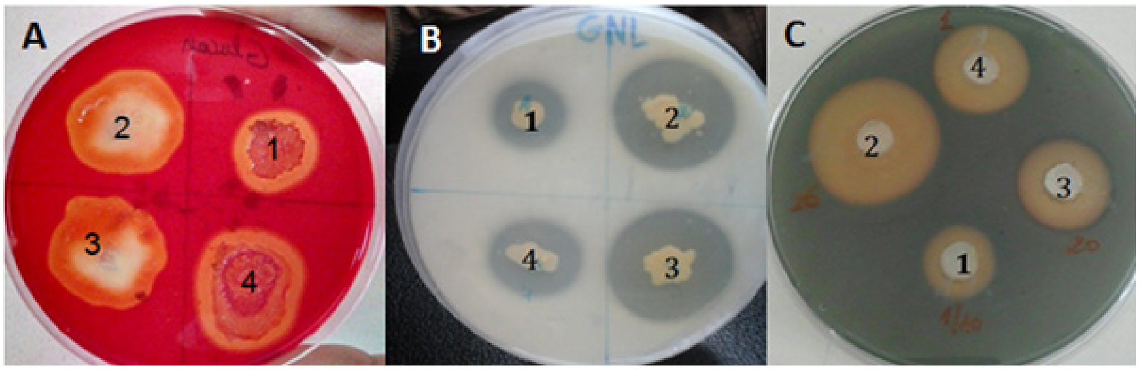

Figure S1: Production of β-glucanase (A), proteases (B) and siderophores (C) *in vitro* by bacterial isolates 1: *Bacillus pumilus* MA9, 2: *Virgibacillus halodenitrificans* MA14, 3: *Bacillus subtilis* MA17 and 4: *Bacillus safensis* MA19 after 7 days of growth at 28 ± 2°C. The formation of halo zone around the colonies shows the positive activity.

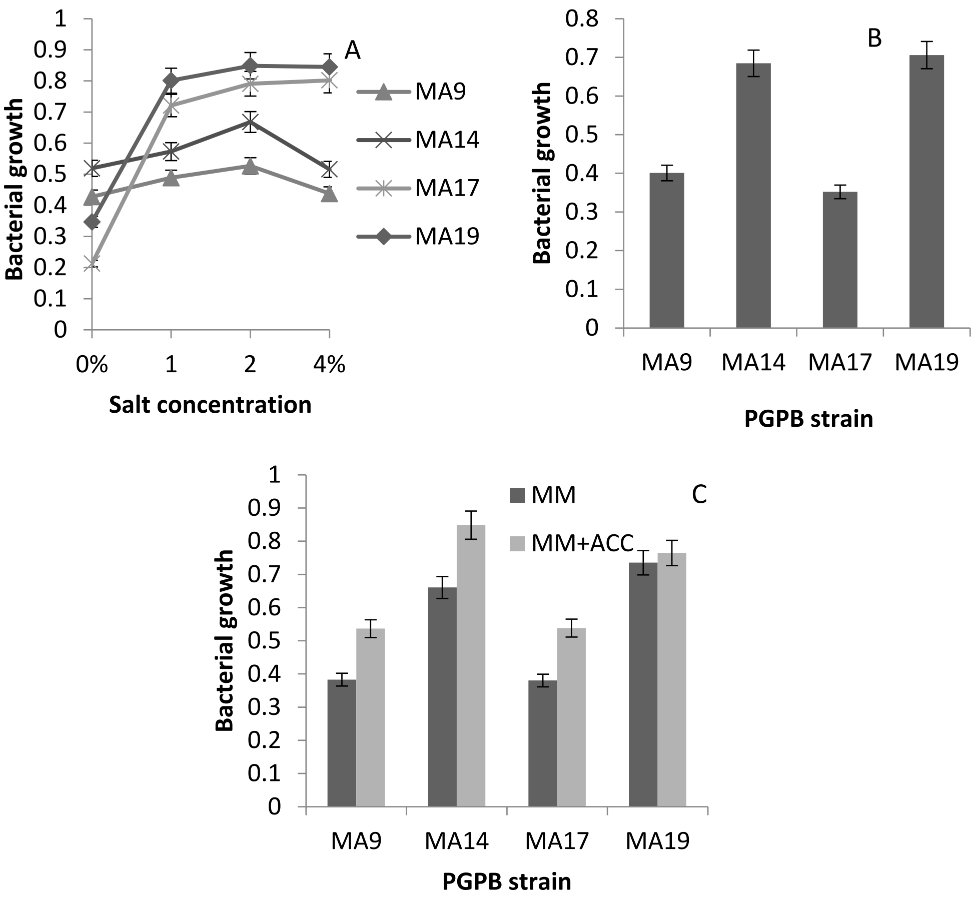

Figure S2 (**A**) Salinity tolerance assay of selected strains on LB medium after 48h with different concentration of NaCl; (**B**) Bacterial growth assay after 7 days of selected bacteria on N free medium (NFMM) and (**C**) Bacterial growth assay after 7 days of selected bacteria on NFMM+ACC as the only available nitrogen source. These three bacterial growth assays were carried out under 30ºC and pH 6.5. Error bars show the standard deviation of the mean values of three replicates ($P<0.05$).

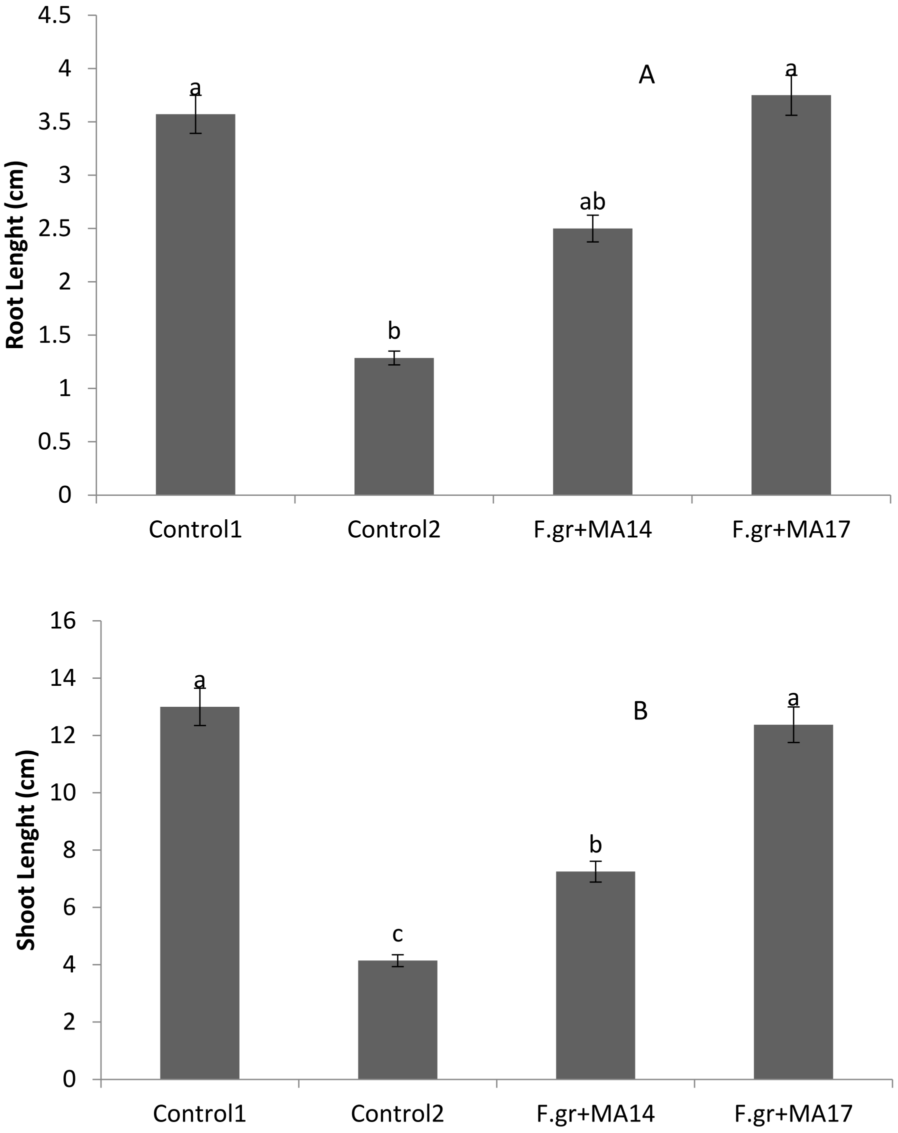

Figure S3 Effect of seed biopriming with PGPB's strains on fusarium wilt suppression expressed in root length (**A**) and shoot length (**B**) following inoculation with *Fusarium graminearum* in seedling explants of durum wheat in a controlled culture chamber.

Control 1 (not inoculated with any microbe), Control 2 (only inoculated with *F. graminearum*), Treatment 1 (inoculated with *MA14* and *F. graminearum*) and Treatment 2 (inoculated with *MA17* and *F. graminearum*). Bars represent standard error of mean (SEM). Values with the different letter within the same column are significantly different at $P < 0.05$ according to Duncan's test.

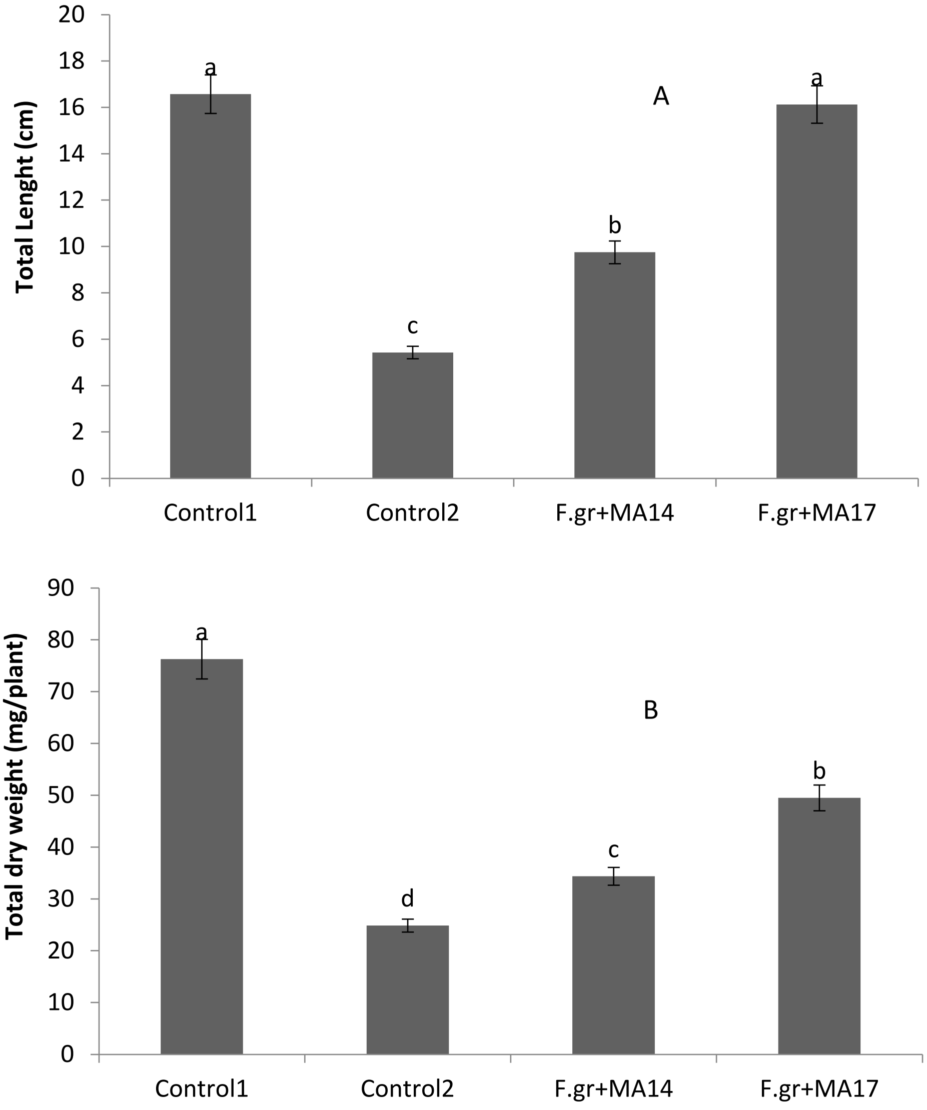

Figure S4 Effect of seed biopriming with PGPB strains on fusarium wilt suppression expressed in total length (**A**) and total dry weight (**B**) following inoculation with *Fusarium graminearum* in seedling explants of durum wheat in a controlled culture chamber.

Control 1 (not inoculated with any microbe), Control 2 (only inoculated with *F. graminearum*), Treatment 1 (inoculated with *MA14* and *F. graminearum*) and Treatment 2 (inoculated with *MA17* and *F. graminearum*). Bars represent standard error of mean (SEM). Values with the different letter within the same column are significantly different at $P < 0.05$ according to Duncan's test.

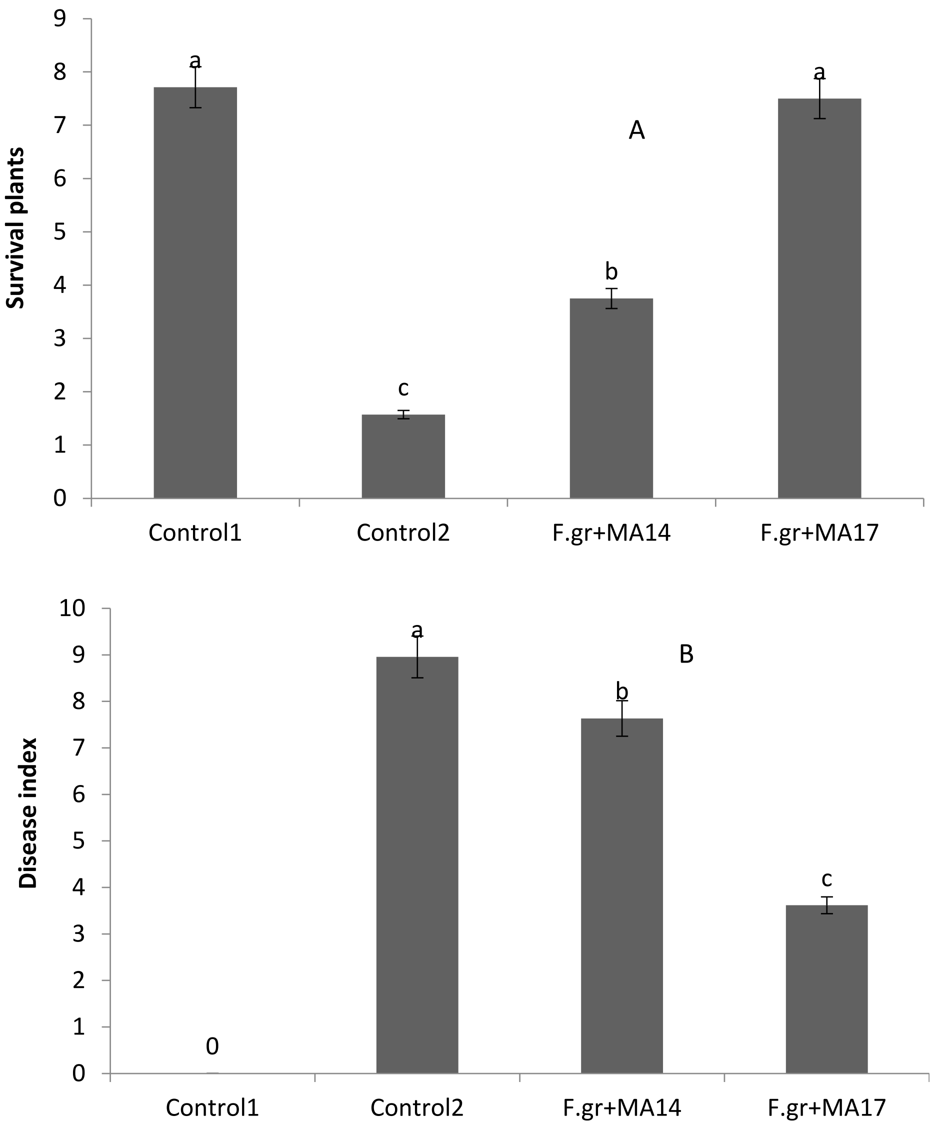

Figure S5 Effect of seed biopriming with PGPB strains on fusarium wilt suppression expressed in survival of explants (**A**) and disease index (**B**) following inoculation with *Fusarium graminearum* in seedling explants of durum wheat in a controlled culture chamber.

Control 1 (not inoculated with any microbe), Control 2 (only inoculated with *F.graminearum*), Treatment 1 (inoculated with *MA14* and *F. graminearum*) and Treatment 2 (inoculated with *MA17* and *F. graminearum*). Bars represent standard error of mean (SEM). Values with the different letter within the same column are significantly different at $P < 0.05$ according to Duncan's test.

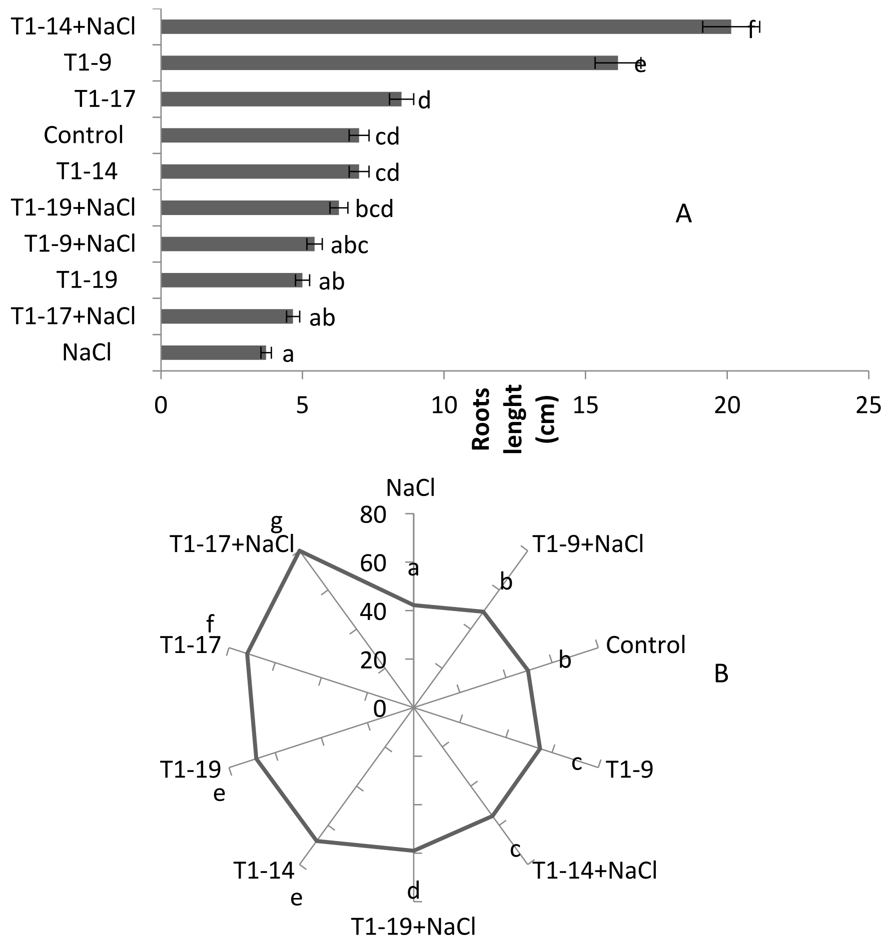

Figure S6 Effect of seed biopriming with PGPB strains on root length (**A**) and total length (cm) (**B**) of durum wheat plants without and with 125 mM NaCl treatment.

Values with the different letter within the same column are significantly different at $P < 0.05$ according to Duncan's test. Control = no NaCl and no PGPB; NaCl = no PGPB in salinized soil; T1 = bioprimed seeds; 9 = *Bacillus pumilus* MA9; 14 = *Virgibacillus halodenitrificans* MA14; 17 = *Bacillus subtilis* MA17; 19 = *Bacillus safensis* MA19.

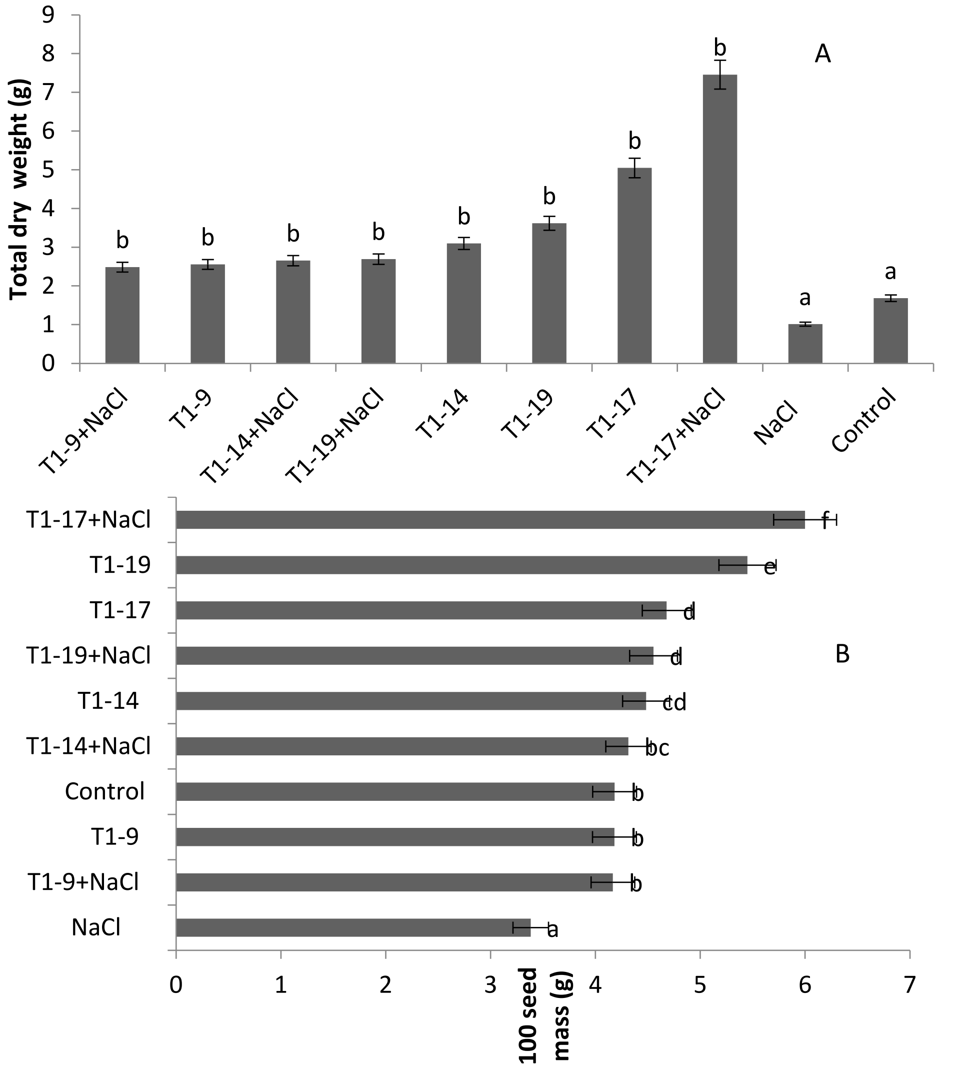

Figure S7: Effect of seed biopriming with PGPB strains on total dry weight (**A**) and 100 seed mass (**B**) of durum wheat plants without and with 125 mM NaCl treatment.

Bars represent standard error of mean (SEM). Different letters above the error bars indicate significant difference at $P < 0.05$. Control = no NaCl and no PGPB; NaCl = no PGPB in salinized soil; T1 = bioprimed seed; 9 = *Bacillus pumilus* MA9; 14 = *Virgibacillus halodenitrificans* MA14; 17 = *Bacillus subtilis* MA17; 19 = *Bacillus safensis* MA19.

.
